# Exploring Barriers, Enablers, and Strategies for Implementing Learning Health System Projects in Healthcare Organizations: A Multilevel Analysis in an Australian Setting

**DOI:** 10.1002/lrh2.70103

**Published:** 2026-07-13

**Authors:** Louise Shaw, Meg Perrier, Kayley Lyons, Mikayla Wolfe, Rahul Barmanray, Daniel Capurro, Wendy Chapman, Sathana Dushyanthen

**Affiliations:** ^1^ Department of Critical Care University of Melbourne Parkville Victoria Australia; ^2^ Emergency Research Team Murdoch Children's Research Institute Parkville Victoria Australia; ^3^ Centre for Digital Transformation of Health University of Melbourne Carlton Australia; ^4^ Centre for Health Policy, Melbourne School of Population and Global Health University of Melbourne Parkville Australia; ^5^ The Royal Melbourne Hospital Parkville Australia

**Keywords:** digital health, healthcare innovation, implementation, learning health systems, organizational change, quality improvement

## Abstract

**Objective:**

To analyze the barriers, enablers, and strategies affecting the implementation of learning health systems (LHS) projects across individual (micro), organizational (meso), and system (macro) levels within Australian healthcare settings

**Methods:**

Semistructured interviews were conducted with 26 clinicians undertaking LHS projects in their organizations, as part of a LHS Academy fellowship program. Interviews were completed at two discrete time points: early in project implementation and again at project completion. They explored fellows' experiences with implementing LHS projects in real‐world organizational contexts. Data were analyzed using a hybrid deductive‐inductive approach guided by a multilevel ecological codebook developed by Shaw et al.

**Results:**

Participants' early interviews highlighted limited confidence, emerging leadership skills, and uncertainty in navigating implementation challenges, while later interviews described greater confidence, stronger leadership capability, and more refined adaptive strategies at the micro level. Organizational influences were described at both time points as slow and uneven, though later interviews included examples of small cultural shifts and early collaborative efforts alongside persistent constraints such as limited resources, siloed cultures, and inadequate digital infrastructure. At the system level, barriers including regulatory complexity, policy misalignment, and limited funding were reported consistently across both interviews and were perceived as largely unchanged.

**Conclusions:**

Building individual capability was necessary but insufficient for successful LHS project implementation. Alignment across micro‐, meso‐, and macrolevels is critical, requiring parallel investment in organizational readiness, digital infrastructure, and policy reform. Fellowship programs may support individual change and foster proof‐of‐concept projects, but scaling impact demands coordinated, multilevel strategies.

## 1 Introduction

Healthcare improvement increasingly depends on health systems' capacity to learn continuously from their own data and experiences. Digital transformation—the process by which healthcare organizations use data, digital technologies, and continuous learning to redesign care processes and improve outcomes—is one important enabler of this capacity, yet its realization depends on how effectively organizations learn from the data they generate [1]. Much of the progress in healthcare occurs through small‐scale quality improvement projects [2]. However, without a system‐level framework to integrate and scale these efforts, their impact remains limited. The learning health system (LHS) offers such a framework, guiding health systems in how to connect localized data‐driven insights with the design, implementation, and evaluation of solutions, enabling continuous learning and improvement at scale [3]. Operationalizing an LHS approach shares features with other innovation models in complex adaptive systems, relying on disciplined experimentation, where organizations “try multiple approaches and let direction arise by gradually shifting time and attention towards those things that seem to be working best” [4]. The core operational unit of a LHS is the locally implemented project, driven by a learning community and evaluated for impact and scalability.

Over the past decade, interest in LHS research and implementation has grown internationally, although systematic reviews show that much of the literature remains conceptual or descriptive, with only a minority of publications reporting empirical studies of LHS implementation processes, outcomes, or evaluation methods [5, 6, 7]. Despite momentum, many healthcare organizations continue to face challenges in implementing LHS initiatives, including redesigning workflows, integrating disparate data systems, navigating governance and ethics, and fostering culture change [7, 8, 9, 10, 11].

The implementation of an LHS is inherently multilevel, requiring coordination across micro (individual), meso (organizational), and macro (system) levels [12] within complex adaptive environments [12, 13, 14]. Achieving alignment across these levels remains a persistent challenge, particularly where local innovation outpaces organizational or policy readiness.

Several frameworks have been developed to guide the establishment and operation of LHSs at an organizational level, yet implementers remain in the early stages of understanding optimal methods for success. Four LHS models have emerged: the participative model, the embedded researcher model, the pragmatic trial model, and the programmatic model [12]. This study focuses on the participative model, which involves conducting improvement cycles targeting specific health problems. Despite the emergence of these models, significant knowledge gaps remain in understanding how LHS projects are implemented in practice, particularly with respect to the barriers, enablers, and strategies used to address them [14, 15, 16]. Much of the existing literature has been high‐level, limiting the ability of implementers to identify context‐specific, actionable approaches [13] and most studies are cross‐sectional, providing static snapshots rather than reflecting the dynamic and iterative nature of implementation work [17].

It is important to distinguish two related but conceptually distinct challenges that run through the LHS literature. The first is LHS establishment: the broad, sustained organizational endeavor of building the infrastructure, culture, governance, and data systems required for continuous cycles of learning and improvement across a health service. The second is LHS project implementation: the discrete, locally‐led improvement cycles that operationalize LHS principles in practice, described by Friedman and Greene as the work of the participative model [12]. These challenges operate at different scales and timescales. Frameworks such as those of Foley and Vale [18] and McLachlan et al. [19] address LHS establishment at an organizational or system level, while questions about the barriers, enablers, and strategies encountered by individuals implementing specific improvement projects remain comparatively underexplored in the empirical literature. This study focuses explicitly on the challenge of project implementation within an LHS context and contributes empirical, longitudinal evidence on the multilevel factors shaping this work.

This study seeks to address these gaps by exploring the barriers, enablers, and strategies influencing the implementation of LHS projects within Australian healthcare organizations, through exploratory interviews with participants in the LHS Academy at two time points [20]. The LHS Academy was a one‐year fellowship program developed by the University of Melbourne in collaboration with Melbourne Academic Centre for Health (MACH) affiliated health services. Practicing health professionals completed a 2‐month intensive foundational course in applied LHS principles. This was followed by 8 months of project‐based learning, whereby the fellows implemented an LHS‐aligned improvement project within their organization [20]. Using a multilevel ecological perspective, this study examined the individual, organizational, and system‐level factors shaping LHS project implementation [21] and provides empirically derived insights to inform policy, guide organizational decision‐making, and bridge the gap between conceptual LHS models and their practical application.

## 2 Methods

### 2.1 Study Design

This study employed a phenomenological qualitative methodology [22] with interviews conducted at two time points to examine barriers and enablers at project commencement and completion. This approach provided insight into how fellows described their experiences at different stages of project implementation. This study adhered to the Guidance for Reporting Qualitative Research in Informatics framework, as outlined by Ancker et al. [23].

### 2.2 Theoretical Framework

Semi‐structured interview guides (Supporting Information 1) were informed by Bronfenbrenner's ecological systems theory (EST) [24] and the consolidated framework for implementation research (CFIR) [25] to sensitize analysis to multilevel contextual influences on implementation. Although the LHS Academy primarily targeted individual capability, fellows' project experiences were strongly shaped by organizational and system‐level factors, making the combined frameworks appropriate for capturing these contextual influences.

### 2.3 Ethics Approval

Ethics approval was obtained from the University of Melbourne's Human Research Ethics Committee (ID: 24432) and informed consent was obtained from all participants.

### 2.4 Setting

The study was conducted within the LHS Academy fellowship program run by the Centre for Digital Transformation of Health at the University of Melbourne from 2022 to 2024 [20]. The LHS Academy was a 1‐year fellowship program designed for practicing health professionals to engage with the LHS model, which provides a cyclic quality improvement framework for health organizations to incorporate the use of routinely collected health data to improve patient care and outcomes. The program consisted of a 2‐month intensive short course on applied LHS principles and core LHS competencies, followed by 8 months of project‐based learning supported by workshops, peer mentorship, and networking activities. Fellows implemented an LHS‐aligned project within their healthcare organization and received clinical and research methods mentorship throughout the program (Supporting Information 2).

### 2.5 Participants and Selection

Participants (Fellows) were healthcare professionals enrolled in the LHS Academy and were from medical (*n* = 12), nursing (*n* = 6), and allied health (*n* = 8) backgrounds from both the tertiary and primary care sectors. Fellows were experienced clinicians (mostly with over 10 years of clinical experience, with medical fellows typically within 2–5 years of completing specialty training), predominantly operating as informal or emerging leaders within their organizations.

Eligibility criteria included employment at ≥ 0.5 FTE at the participating health service, and documented support from line managers and executive leadership to undertake an LHS‐aligned project. Fellows were selected annually by organizational executive leadership in collaboration with the University of Melbourne Centre for Digital Transformation of Health [20]. These criteria reflect recognition of their potential for organizational influence, even if they did not hold formal leadership positions. Prior quality improvement or health services research experience was not required or measured at entry, consistent with Forrest et al.'s recommendation that foundational competencies not be set as prerequisites for LHS training [26]. The fellowship targeted development across Forrest et al.'s domains of improvement and implementation science: engagement, leadership and research management; informatics; and research methods [26].

### 2.6 Recruitment

All 36 LHS Academy Fellows (2022–2024) were invited to participate. Recruitment occurred during program onboarding, with study information and electronic consent provided.

### 2.7 Data Collection

Semi‐structured interviews were conducted online (Zoom) at two time points: entry interviews (2–4 months post commencement) and exit interviews (1–2 months post completion). Interviews lasted 30–60 min and explored expectations, perceived barriers and enablers, implementation strategies, and reflections on project progress at individual (micro), organizational (meso), and system (macro) levels. All interviews were audio‐recorded, transcribed using Otter.ai, and manually reviewed for accuracy and deidentification.

### 2.8 Data Analysis

Analysis was guided by a previously published multilevel codebook for identifying barriers, enablers, and strategies for implementing LHS projects [21]. Interview data were analyzed using a deductive‐inductive content analysis method [27]. Themes were organized across individual, organizational, and system levels, with comparative analysis of entry and exit interviews enabling examination of changes in perceptions over time.

To ensure rigor, two coders (L.S. and M.W.) conducted iterative coding with meetings to resolve discrepancies, maintain reflexivity, and document analytic decisions.

## 3 Results

A total of 26 fellows (out of a cohort total of 36) participated across three cohorts (2022–2024) (Table 1). Entry interviews were conducted with 26 fellows, while project completion interviews were completed with 23 fellows. Response rates were similar for all three cohorts (2022: 9/10 fellows; 2023: 7/9 fellows; 2024: 7/7 fellows). The three noncompletions arose from one fellow leaving the program due to a change in employment, and two fellows were unable to schedule a time within the exit window due to clinical demands.

**TABLE 1:** Summary of fellowship participation and interview completion.

Year	Total number of fellows	Participant ID numbers	Number of entry interviews	Number of exit interviews
2022	10	10–19	10	9
2023	9	27–36	9	7
2024	7	37–43	7	7
Total	26	—	26	23

Tables 2, 3, 4 provide an overview of how fellows' reflections and learning differed between entry and project completion interviews.

**TABLE 2:** Microlevel: Individual capacity building.

Theme	Description of pattern	Representative quotes (entry)	Representative quotes (exit)
1. Skills development and personal attributes			
Stakeholder engagement skills	Among the most frequently discussed skill areas. At the start of the fellowship, fellows spoke about the need to develop these skills; by the end, they described actively applying them to build relationships and influence change.	“A personal barrier for me is my position within the organization. So, I am quite junior and quite green in my role. So therefore, working on a project that has such wide ramifications and involves a lot of stakeholder consultation and decision by people who are much higher up the chain than I, is a challenge and getting them to do work for me.” (ID41) “I feel like I don't have I don't have a lot of power nope, pack a lot of punch. If not, I mean, to get things moving, I need someone higher up to get to get things moving a bit faster and progress things a bit quicker than myself.” (ID14)	“The ongoing engagement with stakeholders and really learning what drives them has been crucial. I've had to work with eight different groups, and identifying their specific pain points and addressing them one by one has been essential in building trust and gaining their endorsement. It's been a huge part of successfully leading this project forward.” (ID17) “Communication and stakeholder engagement has become second nature to me now. In the beginning, I was nervous leading these conversations, but now I feel completely confident sitting down with executives, clinicians, or IT teams to make the case for change. Those relationships havebeen the foundation of our progress.” (ID11)
Time management	A significant early challenge, often described with frustration and overwhelm. By the end of the fellowship, many fellows reported developing strategies to balance competing demands, though pressures were not fully resolved.	“Whilst I had notionally quarantined time to do it, my job has multiple parts, and I've struggled with boundaries around how much time I have to dedicate to the project versus other responsibilities.” (ID32) “Balancing project work withother responsibilities is tricky, especially when timelines shift. It's been about adapting and continuing progresswithout letting other things slip.” (ID39)	“There'd be LHS‐heavy weeks where I'd catch up because I spent my time on EMR or ED or other projects, and LHS‐light weeks. It was about balancing priorities.” (ID43) “I made a plan at the start and tried to stick to those timelines. Even though I had to make some modifications, having the timeline helped me to reach my goals.” (ID19)
Leadership and teamwork skills	Consistently recognized as important throughout the fellowship. Fellows increasingly saw themselves as leaders, shifting from supporting change to actively driving it.	“I recognize that maybe one of my strengths is my ability to work within a team, but leading teams still feels new and a bit intimidating.” (ID22) “I have pre‐existing relationships with key people, but I'm still learning how to leverage those relationships to lead change.” (ID37) “I think my strength isbringing people togetherfor conversations, though I wouldn't call myself a leader yet.” (ID34)	“Being part of a research team was a huge enabler—I now see myself as someone who can lead these efforts rather than just support them.” (ID27) “I can now step into meetings and clearly convey ideas, which has helped me lead our project forward.” (ID33) “I've grown comfortable leading difficult conversations and helping others navigate complex decisions.” (ID 11)
Implementation skills	Continuously discussed across both interviews, showing that practical implementation knowledge remained a core area of focus that required ongoing attention.	“I don't like to let people down, but I sometimes lack the practical skills needed to implement these changes.” (ID29) “My organizational skills are good but applying them to research implementation is still new.” (ID35) “I'm confident clinically, but I'm less certain about how todrive implementation steps.” (ID 39)	“We've been putting in business cases and redesigning workflows—I've learned so much about practical implementation.” (ID41) “I now know how to integrate implementation planning into everyday workflows.” (ID25) “Seeing my project through to real‐world application has been the most rewarding part.” (ID29)
2. Mindset and confidence			
Confidence (barrier)	Initially a major barrier, with many fellows expressing self‐doubt and hesitation. By the end, these concerns had significantly diminished.	“Half the time I'm not sure what everyone's talking about — it feels overwhelming.” (ID17) “I sometimes fear failure and worry I'm not experienced enough for this role.” (ID22) “At the start, I wasn't sure I belonged here or had the right skills.” (ID28) “I worried about letting myteam down because Ididn't know enough.”(ID30)	“By the end, I felt capable of speaking up and contributing confidently in meetings.” (ID33) “I now trust my judgment and feel ready to lead future projects.” (ID38) “My confidence has grown hugely—I now approach problems with clarity instead of hesitation.” (ID19) “I'm no longer afraid to challenge decisions or offer solutions.” (ID22)
Confidence‐building strategies	Showed the greatest change across the two interview points, reflecting the proactive steps fellows took to build resilience and capacity to lead change.	“Right now, my approach to building confidence is mostly about watching and learning from others. I observe how senior colleagues handle complex situations and try to model their behavior, but it still feels external and a bit tentative.” (ID24) “Joining the fellowship was a deliberate step toward challenging myself. I wanted to push outside my comfort zone anddevelop the resilience I knowI'll need for leadership roles.” (ID26)	“Over time, I've developed clear strategies for building and maintaining confidence. Seeking out mentors has been invaluable, as has breaking big challenges down into manageable steps so I don't feel overwhelmed.” (ID34) “Regular reflection has been a key tool for me—it allows me to recognize my growth and identify areas to focus on, which reinforces my confidence.” (ID36)
Self‐reflective capacity	Initially recognized as important but largely theoretical. By exit, reflection was applied in practice, with fellows describing how it shaped their decision‐making and growth.	“Reflection is something I know is important, but it feels quite abstract to me at this point. I don't really have a structured way of doing it, so I tend to reflect only when something goes wrong.” (ID19) “I sometimes think about lessons learned, but I haven't developed a consistent process for reflection that feeds back into how I work.” (ID25)	“I now build reflection into my weekly schedule. It helps me step back from the day‐to‐day and think strategically about what's working and what needs to change. It's been transformative for my decision‐making.” (ID35) “Reflecting on my growth throughout the fellowship has been incredibly empowering. It's helped merecognize not just the challengesbut also the progress I've made.”(ID40)
Positive attitude toward innovation	Many fellows began with enthusiasm for innovation, which became more focused and pragmatic by the end of the fellowship, reflecting realistic understanding of implementation challenges.	“I'm genuinely excited about the potential of everyone's projects. There's so much energy and enthusiasm in this group, and it feels like we're on the cusp of making meaningful change.” (ID18) “I love working with new ideas and technologies, even when I don't fully understand how they'll play out in practice. It's both exciting and a little scary.” (ID25)	“My enthusiasm for innovation is still strong, but it's now tempered by a realistic understanding of the challenges involved. I've learned that true innovation requires patience and persistence.” (ID37) “I've come to see innovation as a balance between ambition and practicality. It's not about flashy new projects, but about embedding change in a way that lasts.” (ID22)
3. Strategic adaptation and support‐seeking			
Seeking support	At entry, support‐seeking was broad and undefined. Over time, it became more strategic and purposeful, with fellows identifying specific sources and types of support that would help their work.	“At the beginning, I knew I would need support, but I wasn't really sure where to find it or what exactly to ask for. It felt like there were lots of people who might be able to help, but no clear pathway to connect with them or to even know what type of support would make the biggest difference.” (ID28) “Sometimes it felt like shouting into the void—I'd reach out for help, but I wasn't sure who the right person was or whether I was even asking the right questions. It was very trial and error.” (ID32)	“By the end of the year, I had a much clearer understanding of who I could approach for specific issues—whether that was data, workflow, or stakeholder engagement. Instead of just asking for help generally, I now go in with a targeted request and a clear idea of what I need.” (ID40) “I've built a strong network of people who I know I can rely on. It's no longer about finding support by chance—it's about drawing on trusted relationships strategically.” (ID42)
Collaboration and networking skills	The most frequently discussed strategy at both entry and exit. While focus decreased slightly, networking remained a central and enduring enabler of LHS success.	“Networking feels overwhelming—there are so many potential connections to make, and it's hard to know where to start or how to build relationships that will actually help the project.” (ID19) “I've made a few connections through the fellowship, but it's been mostly opportunistic rather than planned. I know collaboration is important, but I'm still figuring out how to do it well.” (ID23)	“Networking has become second nature to me now. I proactively reach out to people across departments, and those relationships have been key to getting things done. It feels purposeful rather than accidental.” (ID35) “Collaboration has really taken off—we now have cross‐departmental teams working on shared goals. It's incredibly rewarding to see silos breaking down.” (ID38)
Protecting time for LHS work	Fellows described a move toward protecting time for improvement activities, though this remained challenging due to external demands.	“I don't have any dedicated time for this work—it's constantly competing with my clinical and administrative duties. Some weeks, it feels almost impossible to make progress because there's always something more urgent pulling me away.” (ID30) “Finding space for improvement work feels like squeezing water from a stone. Even when I block time in my calendar, emergencies and last‐minute requests always seem to take over.” (ID31)	“I've managed to carve out small windows of protected time for project work. It's still a challenge, but even a few consistent hours each week have made a noticeable difference.” (ID42) “Balancing competing priorities remains difficult, but I've become better at negotiating for protected time and defending it when needed.” (ID44)

**TABLE 3:** Mesolevel: organizational context.

Theme	Description of pattern	Representative quotes (entry)	Representative quotes (exit)
1. Resource constraints			
Funding limitations	A persistent barrier throughout the year. Fellows developed creative strategies to work within constraints, but the underlying issue remained unresolved.	“Our biggest limitation right now is funding. We have so many innovative ideas and a motivated team, but without resources to implement them, they remain concepts on paper. It's incredibly frustrating to design something that we know will improve patient care and then have to shelve it because there's no budget to make it happen.” (ID12) “Funding streams are opaque and inconsistent. Even small costs like printing or licenses become big hurdles when there's no clear pathway to secure funds, which makes planning feel almost impossible.” (ID16)	“We've found creative ways to keep things moving, like sharing resources across departments or piggybacking on other projects, but these are temporary fixes. The fundamental funding gap hasn't changed—it's still the biggest barrier to scaling our work.” (ID33) “We've learned to align our funding proposals with organizational priorities, which has helped us get some approvals through, but it's still a constant battle. Each success feels fragile.” (ID 18)
Time constraints	Initially a major issue for both individuals and teams. While some local improvements were reported, time pressures remained a substantial barrier by exit.	“Clinicians want to participate, but their schedules are already packed with clinical duties. It's heartbreaking to see opportunities for improvement stalled because there's literally no room in the calendar.” (ID14) “Time is the biggest barrier—we can buy equipment or hire consultants, but we can't create more hours in the day for clinicians to engage with these projects.” (ID 26)	“We've made some progress in negotiating small blocks of protected time, but it's still a constant juggling act. The lack of time continues to slow down every stage of implementation.” (ID39) “We've introduced weekly time slots dedicated to project work, which has been a game changer for team focus, even though they're often interrupted by urgent clinical issues.” (ID 20)
2. Organizational culture and change			
Cultural resistance to change	A prominent theme at entry that decreased considerably by exit, reflecting growing acceptance of new approaches in some contexts.	“Some staff are deeply skeptical about why we need to change existing processes. There's a sense of, ‘we've always done it this way,’ and challenging that mindset can feel like pushing a boulder uphill.” (ID12) “Resistance often comes from fear—fear of losing control, fear of making mistakes, or fear that the project will create extra work without real benefits.” (ID24)	“We've seen a noticeable shift in attitudes. Staff who were initially skeptical are now cautiously supportive, especially after seeing small but meaningful improvements.” (ID37) “People are now coming to us with their own suggestions for change, which is a huge turnaround from where we started.” (ID22)
Supportive learning cultures	Initially discussed as aspirational. By exit, some fellows described early steps toward fostering organizational learning, though changes were often localized and fragile.	“A culture of learning would transform how we work, but right now, it just doesn't exist. Reflection and improvement happen sporadically, if at all, and mostly when something has gone wrong.” (ID16) “We need safe spaces where staff can test new ideas without fear of blame or retribution. At the moment, mistakes are hidden instead of being used as learning opportunities.” (ID23)	“We've started to see small pockets of learning culture emerge, like regular debriefs and shared reflection sessions. It's still fragile, but it's a promising sign.” (ID39) “These initiatives are helping people see that improvement is everyone's responsibility, not just something done by a select few.” (ID42)
Collaborative working practices	Participants described moderate progress in teamwork and cross‐team collaboration but noted it remained an ongoing focus requiring sustained attention.	“Departments operate like separate kingdoms. Collaboration happens only when there's a crisis, not as a matter of routine.” (ID13) “Collaboration depends on who knows who rather than on systems or structures. If a key person leaves, the whole connection falls apart.” (ID24) “We lack any formal processes for joint problem‐solving, which makes complex projects incredibly difficult to manage.” (ID28)	“We now have regular cross‐departmental meetings, which are helping to break down silos and create shared ownership of projects.” (ID38) “Collaboration is still fragile, but there's a sense that people want to work together rather than just protect their own turf.” (ID40)
3. Technology and infrastructure			
Data availability and quality	A persistent challenge, with isolated instances of improvement noted in a few sites but no widespread change.	“Our data is messy and incomplete. It's hard to trust it for decision‐making, which means we're often working blind when planning interventions.” (ID14) “Collecting reliable data is like pulling teeth. Clinicians don't always enter it consistently, and there's little incentive for them to do so.” (ID23)	“We've made some progress, like standardizing a few fields, but there's still a long way to go before we have truly reliable, actionable data.” (ID39) “Some gaps are closing slowly, but overall, data quality remains one of our biggest barriers.” (ID21)
EMR limitations and interoperability issues	Fellows consistently reported frustration with system incompatibilities and lack of integration, which remained unresolved by the end of the fellowship.	“Our EMR doesn't integrate with other systems, so staff end up duplicating work across multiple platforms. It's inefficient and deeply frustrating.” (ID12) “Integration issues slow down even routine tasks. Clinicians feel like they're fighting the system rather than being supported by it.” (ID22)	“There have been some small improvements, but the core problems remain. Systems still don't talk to each other, and staff are left to bridge the gaps manually.” (ID38) “There's been virtually no progress on interoperability. We're still dealing with the same frustrations we had at the start.” (ID25)

**TABLE 4:** Macrolevel systems context.

Theme	Description of pattern	Representative quotes (entry)	Representative quotes (exit)
1. Policy, regulation, and funding			
Legal and ethical issues	Participants consistently raised concerns about regulations and governance, noting limited system‐level support in these areas.	“Navigating the regulatory landscape feels like moving through treacle. Every step of the project requires multiple layers of approval, and the processes are slow and unclear. It often feels like the system is designed to stop progress rather than support it.” (ID11) “Policies aren't aligned across organizations. Something approved in one health service might be rejected in another, creating a maze of confusion and delays.” (ID23)	“The same regulatory hurdles are still there. We've become more skilled at navigating them, which makes the process feel slightly smoother, but the fundamental barriers haven't changed at all.” (ID36) “Regulatory complexity continues to be one of the most significant barriers to scaling innovation. It's like playing a game where the rules change constantly and no one tells you why.” (ID25)
System‐level funding limitations	Fellows noted some small signs of improvement, but sustainable funding remained a critical challenge for LHS growth.	“There is no dedicated funding stream for learning health systems work. Every dollar we use has to be cobbled together from different sources, which makes planning long‐term projects almost impossible.” (ID13) “Funding for long‐term infrastructure simply doesn't exist. We get small wins here and there, but nothing that allows us to invest in real, transformative change.” (ID21)	“There have been some early conversations about creating dedicated funding streams, but so far, it's all talk and no action. We're still piecing things together in the same ad hoc way.” (ID38) “We've seen tiny improvements, like slightly larger grants, but nothing transformative. It's frustrating to keep operating in survival mode.” (ID24)
2. Collaborative networks			
Collaborative networks as enablers	Initially described with optimism and hope. By exit, some early progress was visible, but networks were still fragile and in development.	“We talk about building networks across organizations, but right now, it's mostly just an aspiration. There's no formal structure or dedicated resources to make it happen.” (ID12) “There's excitement about working together across services, but nothing concrete has been set up yet. Everyone agrees it's a good idea, but no one owns it.” (ID20)	“We've begun small‐scale collaborations that are showing real promise. They're still fragile and dependent on a few key people, but it's a step forward from where we started.” (ID37) “Collaborative networks are slowly starting to take shape. There's now a sense of shared purpose, even if the systems to support it aren't fully there yet.” (ID23)

### 3.1 Microlevel Results: Individual Capacity Building

Many of the enablers identified by fellows were at the individual level, reflected in increased confidence, strengthened leadership skills, and the development of adaptive strategies to overcome barriers (Table 2).

#### 3.1.1 Skills Development and Personal Attributes

Fellows described noticeable growth in core LHS competencies across the fellowship period. These competencies, consistent with those identified by McDonald et al. [28] and Forrest et al. [26] encompass: implementation science and quality improvement methods; data access, analysis, and interpretation; stakeholder engagement and communication; project management; and leadership for change. The LHS Academy intentionally targets development of these competencies through its foundational course, mentorship structures, and project‐based learning; fellows were not required to demonstrate these competencies at entry, with eligibility centred on clinical experience, organizational support, and a project idea. Fellows entered the program with strong clinical skills, but many participants reported lacking the necessary skills to manage projects, engage diverse stakeholders, and interpret data effectively. Early stakeholder interactions were tentative, with uncertainty about who to involve or how to gain buy‐in. Discussions about stakeholder engagement were often framed in terms of what was missing or what they “wished they could do.” By exit interviews, these same conversations shifted toward descriptions of how these skills were being applied in practice, with fellows sharing concrete examples of building partnerships and facilitating cross‐disciplinary collaboration.

Time management was a frequent concern at entry, reflecting the challenge of balancing fellowship responsibilities with ongoing operational duties. Fellows frequently described feelings of overwhelm and frustration about competing demands. By exit, these concerns had decreased substantially, and participants reported that they had developed structured approaches to prioritizing their LHS activities and negotiated protected time with colleagues and supervisors.

Leadership and teamwork skills were consistently recognized as foundational skills for implementing LHS projects, and many fellows increasingly reflected that their skills improved over the course of the fellowship. Reporting of specific implementation skills remained steady over the two time points and was identified as a core competency area requiring ongoing professional development.

#### 3.1.2 Mindset and Confidence Evolution

The largest change occurred in themes related to confidence and adaptive mindset. At entry, a lack of confidence was a frequently reported barrier, with many fellows questioning their ability to drive change or influence decision‐making processes within their organizations. By the end of the fellowship, fellows described themselves as more confident. They also articulated specific strategies they had developed to build and sustain their confidence, such as seeking mentorship, celebrating small wins, and reflecting on progress.

Similarly, capacity for self‐reflection developed throughout the fellowship. At entry, participants often described reflection as an abstract concept or external requirement. By exit, fellows described actively integrating reflective practices into their daily work and using them to guide decisions, evaluate progress, and adjust strategies.

#### 3.1.3 Strategic Adaptation and Support‐Seeking

Fellows' strategies for seeking and using support changed over the course of the fellowship. Initially, participants recognized the need for help in broad and nonspecific terms. By the end of the fellowship, help‐seeking behaviors became targeted and strategic, with fellows able to clearly identify what types of support were most valuable and how to access them.

Collaboration and networking skills emerged as a dominant theme throughout the fellowship. While the emphasis on this strategy was less frequently described in the exit interviews, it remained the most frequently cited microstrategy. Fellows moved from seeing networking as a task to viewing it as a foundational practice for success.

### 3.2 Mesolevel Results: Organizational Context and Persistent Barriers

Organizational factors were characterized by small but variable shifts in culture and collaboration, with structural barriers such as time, funding, and data availability consistently reported at both entry and exit (Table 3).

#### 3.2.1 Resource Constraints as Persistent Barriers

Organizational barriers proved more resistant to change than individual‐level factors. Resource limitations, particularly funding and staffing, were consistently identified as major barriers at both interviews. In some cases, these constraints were described as intensifying, creating ongoing frustration for fellows. While some fellows were able to negotiate time and space for LHS activities, time pressures remained a substantial organizational barrier, reflecting the tension between improvement work and operational demands.

#### 3.2.2 Organizational Culture and Change

Themes related to organizational culture showed greater variation between entry and exit interviews. At entry, fellows frequently discussed the importance of organizational environments that support learning and innovation but also described barriers such as cultural resistance to change and skepticism among colleagues. By exit, some fellows described a perceived reduction in cultural resistance, citing examples of previously skeptical colleagues becoming more engaged or supportive. However, it cannot be ruled out that this reflects fellows' adaptation to or normalization of persistent resistance, rather than its actual reduction. Fellows additionally noted enablers such as emerging pockets of learning culture, increased cross‐departmental collaboration, and cautious openness to change; these shifts were characterized as localized, fragile, and dependent on key individuals rather than embedded in broader organizational change.

#### 3.2.3 Technology and Infrastructure

Digital infrastructure challenges were reported across both time points. Fellows repeatedly highlighted limited data availability and poor interoperability between systems, including electronic medical records (EMRs). These issues showed little perceived change, reflecting the longer timelines required for system‐wide digital upgrades compared to individual skill development.

### 3.3 Macrolevel Results: Systems Context

At the macrolevel, fellows consistently described complex ethics, regulation and governance, inconsistent policies, and a lack of alignment between governance structures and LHS goals (Table 4).

Some collaborative networks were described as beginning to form during the fellowship, providing optimism about future opportunities for large‐scale coordination. However, these networks remained informal, time‐bound, and limited in scope, without the resources, structural support, or policy backing needed to drive meaningful system‐level change.

### 3.4 Practical Implications for LHS Implementation Across System Levels

Table 5 summarizes the practical implications for implementing LHS initiatives across system levels, highlighting the interconnected barriers, enablers, and strategic actions identified in this study. At the fellowship and capability‐building level, the recommendations emphasize the importance of organizational readiness, structured executive sponsorship, protected time for LHS activity, and explicit training in change management. These factors collectively enable fellows to operate effectively within complex organizational environments.

**TABLE 5:** Practical implications for LHS implementation across system levels.

Recommendation	Description	Expected outcomes
*Fellowship programs and capacity‐building initiatives*		
Organizational readiness assessment	Conduct preprogram evaluation to ensure fellows enter environments with minimum foundational support for LHS work	Better alignment between individual readiness and organizational capacity
Executive sponsor engagement	Require formal organizational commitment with structured touchpoints throughout fellowship to maintain leadership awareness and commitment	Sustained organizational support beyond initial enthusiasm
Continuous mentorship	Ongoing mentorship from leaders who understand how to navigate complex governance structures and systems (e.g., ethics, data access)	Avoid unnecessary project delays and improve time efficiency
Protected time negotiation	Provide fellows with frameworks and support for securing dedicated time allocation as core program activity	Reduced competition between LHS work and operational demands
Cohort‐based learning on change management	Explicitly address organizational change management, not only technical LHS competencies	Fellows equipped to navigate organizational barriers effectively
Extended timeframes or alumni support	Acknowledge mismatch between individual development timelines (1 year) and organizational change timelines (multiple years) through alumni networks or extended programs	Sustained impact beyond fellowship completion; reduced fellow frustration and burnout
Assemble a learning community	At project initiation, adequate time should be spent on identifying the right stakeholders and expertise to engage in a learning community around the problem of interest	Ensures that the right people are consulted on the project, the right problem is being solved, the problem is scoped appropriately and that all perspectives are considered.
*Healthcare organizations*		
Digital infrastructure development	Treat as foundational prerequisite, not afterthought; organizations at “Basic” digital maturity face compounded challenges individual ingenuity cannot overcome	Foundation for data‐driven learning and improvement; reduced technology‐related barriers
Dedicated funding streams	Move beyond external grants or discretionary efforts; create sustainable internal funding for LHS work	Financial sustainability for LHS initiatives; reduced reliance on time‐limited project funding
Organization‐wide culture change initiatives	Extend beyond isolated projects to organization‐wide transformation including leadership development, team‐based structures, and celebration of learning from successes and failures	Embedded learning culture; reduced resistance to change; organization‐wide rather than localized transformation
Realistic implementation timelines	Acknowledge slow pace of organizational change; avoid ambitious short‐term projects lacking sustainability	Sustainable change rather than short‐lived enthusiasm; realistic expectations
Strategic alignment with organizational priorities	Ensure LHS projects connect to organizational priorities and receive visible executive support	Organizational commitment; reduced perception of LHS as “extra work”
*Health systems and policymakers*		
Regulatory streamlining	Reduce bureaucratic burden; create clearer, more consistent pathways for data sharing, ethics approval, and cross‐organizational learning	Reduced administrative barriers; faster implementation cycles
Sustainable funding mechanisms	Dedicate funding to LHS infrastructure and capability development beyond short‐term project funding; recognize that current funding models perpetuate fragility	System‐wide investment in LHS capability; reduced funding uncertainty
Interoperability standards and enforcement	Move beyond aspirational frameworks to practical implementation support and accountability mechanisms	Seamless data exchange across systems; reduced siloed information
Standardized and structured data entry	Mandate standardized, structured data‐entry requirements across health services to improve data quality and interoperability.	This ensures that data becomes streamlined and reduces duplicative work of data cleaning initiatives
Formal collaborative learning networks	Establish formal structures with dedicated resources and policy backing, not relying solely on informal professional relationships	Sustained cross‐organizational learning; knowledge sharing at scale
Targeted digital maturity investment	Prioritize investment for organizations at earlier maturity stages to create equitable capacity for LHS implementation across the system	Reduced variation in digital capability; more equitable foundation for LHS implementation
Accredit digital health roles	Demonstrates to individuals that there is a recognized career path and trajectory to follow	More staff that are enthusiastic about undertaking transformation

At the organizational level, participants described that foundational digital infrastructure, sustainable funding models, realistic implementation timelines, and organization‐wide culture change are critical enablers, with gaps in these areas functioning as major barriers to progress. Finally, at the health system and policy level, the table outlines the need for regulatory streamlining, interoperable data systems, dedicated system‐level funding, and formalized cross‐organizational learning networks to mitigate structural barriers that currently slow or constrain LHS implementation.

Together, these multilevel recommendations demonstrate that successful LHS implementation requires coordinated action across individuals, organizations, and the broader health system, with alignment across these levels emerging as a key determinant of sustainability and impact.

## 4 Discussion

### 4.1 Summary of Core Findings

This study examined barriers, enablers, and strategies for implementing LHS projects within Australian healthcare organizations and identified a consistent pattern: substantial individual capability development occurred alongside persistent organizational and system‐level constraints. Fellows in the LHS Academy reported notable growth in confidence, leadership capacity, and adaptive strategies over their fellowship year, yet organizational and system‐level constraints were perceived as highly resistant to change. This differential rate of change across micro‐, meso‐, and macrolevels highlights a critical implementation gap in which individual readiness appears to outpace organizational and systemic capacity for transformation.

By incorporating two interview time points, this study extends the largely cross‐sectional LHS literature [5, 7, 13, 17] by illustrating how participants' experiences and strategies evolved over time. While individual capacity‐building was essential, it was insufficient in isolation, as structural impediments such as limited funding, constrained digital infrastructure, regulatory complexity, and competing priorities consistently limited the translation of individual capability into sustained organizational impact.

### 4.2 Multilevel Patterns

#### 4.2.1 Microlevel: Rapid Individual Development

The fellowship structure supported individual development through protected time, structured mentorship, and peer learning [20]. Fellows entered with strong clinical expertise but limited experience in implementation science, stakeholder engagement, and data‐driven improvement. Across the fellowship, participants described a transition from self‐doubt toward increased confidence and leadership capability, reflecting their growing familiarity with leading complex change initiatives. Having the confidence to collaborate and network, consistently shown in reviews to be among the most powerful skills in healthcare educational acquisition [29], was repeatedly emphasized. Strategic networking emerged as a purposeful rather than an ad hoc activity, reflecting the importance of communities of practice in LHS implementation [30, 31].

Time management, initially described with frustration and overwhelm, became more structured and intentional, although external pressures persisted. The cultivation of self‐reflective capacity represented a particularly meaningful shift, with reflection evolving from an external requirement to an internalized practice guiding decision‐making and continuous improvement. These findings align with McDonald et al.'s identification of core LHS competencies [28], extending this work by illustrating how participants described the development of these competencies in practice and their interaction with contextual factors. However, participants also emphasized that individual skill development alone was insufficient without supportive organizational and system conditions [32]. Because baseline competencies were not formally assessed, the growth fellows described should be understood as developing through project stewardship, that is, the experience of leading an LHS‐aligned project with mentorship, rather than from a standardized starting point. Fellows from medical, nursing, and allied health backgrounds entered with varied exposure to quality improvement and implementation science, and their trajectories likely reflect this heterogeneity as much as the program itself.

#### 4.2.2 Mesolevel: Partial Progress and Persistent Barriers

Organizational level change was perceived as slower and more variable than individual development, consistent with prior reviews [28]. Some participants reported reduced cultural resistance to change over the fellowship year, though these shifts were often characterized as fragile, person‐dependent, and not yet embedded in routine organizational practice [13, 16]. Where leadership support was visible and early project successes were communicated, participants described a shift in organizational attitudes from skepticism to cautious support. However, these cultural changes typically remained localized to specific teams or units, reflecting the absence of broader, organization‐wide transformation. Empowered leadership has been shown to achieve organizational‐level change in Australian healthcare settings [33]; fellows in the present program may be well positioned to contribute to such leadership roles in the future.

Resource barriers, including funding, time, and staffing, persisted, despite fellows' adaptive strategies, reinforcing evidence that such barriers require dedicated organizational investment rather than individual workaround solutions [15, 17, 34].

Fellows repeatedly reported challenges with data quality, EMR limitations, and poor interoperability, issues well documented in the literature yet unresolved in routine practice [35, 36]. This mismatch between individual readiness and organizational infrastructure underscores the need for coordinated investments in both digital maturity and human capability, as highlighted in recent digital health maturity assessments in Australia [37]. Even in digitally mature organizations, participants highlighted the need for sociotechnical infrastructure and governance to support learning cycles, consistent with prior LHS research [38].

The persistence of organizational barriers despite fellows' adaptive and increasingly sophisticated strategies suggests the importance of assessing and developing organizational readiness in parallel with individual capability‐building. This finding aligns with Foley and Vale's framework highlighting readiness as a central determinant of implementation success [18]. Fellows' adaptive strategies, such as creative workarounds, strategic networking, and incremental approaches, enabled progress but could not fully overcome entrenched structural limitations. Global experiences of LHS implementations similarly highlight the emergence of innovation from necessity demonstrated by the fellows [39].

#### 4.2.3 Macrolevel: Minimal Systems Change

Participants consistently described the fellowship timeframe as insufficient for observing meaningful system‐level change. Consistent with existing literature, ethics and governance processes, and regulatory complexity, exacerbated by Australia's federated health system, were perceived as significant impediments to LHS implementation [17, 28, 40].

While informal collaborative networks began to emerge, participants characterized these networks as fragile and under‐resourced, lacking the policy support and structural arrangements required to drive substantial system change [12, 16].

### 4.3 Contextual Considerations: Digital Maturity and Implementation Readiness

Digital maturity emerged in participants' reflections as a cross‐cutting factor influencing implementation across system levels. This finding has important implications for scaling LHS implementation: adequate digital infrastructure appears to function as a prerequisite rather than a parallel development pathway [1, 17, 35, 36]. Participants highlighted that organizations attempting LHS work without foundational digital systems face compounded challenges that individual capacity‐building alone cannot resolve. However, participants also noted that cultural readiness and effective leadership are as critical as digital maturity in enabling LHS progress [11]. Growing support for LHS innovation in the future will require closer alignment with existing initiatives such as quality improvement and value‐based care models, strengthened collaboration between health services, health services researchers, clinical researchers, and implementation scientists [41]; and more diverse funding streams to support the sociotechnical infrastructure underpinning successful LHSs.

Post‐pandemic system pressures, including workforce shortages and operational demands, further constrained organizational capacity for improvement, reinforcing the need for disciplined experimentation within complex adaptive systems [4].

### 4.4 Comparison With Existing Literature

Our findings substantially extend the predominantly cross‐sectional LHS literature [5, 6, 7] by providing insight into how participants described their implementation experiences at two different stages of the fellowship, thereby illuminating evolving challenges and strategies that are not visible in single‐timepoint studies. While previous systematic reviews by Ellis et al. and Enticott et al. identified barriers and enablers to LHS implementation, they noted limited evidence on strategies to overcome identified barriers [10]. Our study addresses this gap by documenting the approaches fellows reported using and their perceived effectiveness, distinguishing between strategies that appeared to mitigate individual‐level barriers (largely successful) and those targeting organizational and system‐level constraints (perceived as largely insufficient) [9, 14].

McDonald et al.'s scoping review identified requisite LHS competencies but did not examine how competencies develop or interact with organizational context [28]. Our findings extend this work by illustrating that while participants reported developing competencies in stakeholder engagement, project implementation, and data‐driven decision‐making, the translation of these competencies into organizational change was often constrained by persistent structural barriers. This highlights that competency development, while essential, is insufficient in the absence of organizational and system‐level support.

The participative model of LHS project implementation, which underpins the LHS Academy program, emphasizes conducting improvement cycles targeting specific health problems, supported by sociotechnical infrastructure and co‐creation across stakeholders. Our findings suggest that while this model supports individual capability development and enables small‐scale project progress, scaling impact requires more explicit attention to organizational readiness, resource availability, and alignment with system‐level policy and governance processes—elements that may be underdeveloped in current applications of the participative model.

### 4.5 Practical Implications

Fellowship programs like the LHS Academy effectively build individual capability but should be complemented by parallel organizational development efforts. The value of a multilevel approach lies in providing a holistic understanding of the factors participants perceived as influencing LHS implementation and illustrating how these factors interact across system levels. Insights from this study can inform future LHS initiatives by highlighting where different forms of support may need to be directed—with individual capacity‐building yielding relatively rapid perceived benefits, while organizational and system‐level change require longer‐term, more intensive investment.

Participants' accounts reinforce that a certain level of digital infrastructure and organizational readiness are prerequisites for conducting LHS projects. This includes enabling data infrastructure, streamlined data access, structured and high‐quality data collection processes, effective change management support, and a culture that values data‐driven continuous learning and improvement (Table 5). Without these foundations, some projects cannot progress regardless of individual capability.

The organizational needs identified through the fellows' projects can also act as a bottom‐up impetus for the system‐level changes required for project success, such as improved data interoperability or the ability to trial and fund new models of care. This suggests that programs like the LHS Academy would benefit not only from formal sponsorship by healthcare leaders but also from more active involvement of roles such as Chief Medical Information Officers and Chief Quality Officers, who are positioned to align fellows' needs with organizational strategy, digital planning, and governance processes.

### 4.6 Limitations

This study has several limitations. Although interviews were conducted at two time points, the design does not constitute a longitudinal evaluation. The findings reflect participants' perceptions at different stages of their project work and cannot be used to infer causal change over time. Qualitative data were drawn from a purposive sample of fellows who may have been more engaged or motivated than nonparticipants, limiting the representativeness of the findings. Prior quality improvement, implementation science, and research competencies were not measured at entry, so the reported growth reflects perceived development through project stewardship rather than standardized gains attributable solely to the program.

The study was conducted primarily within the Victorian public health system, where a decentralized health service structure and fragmented data infrastructure create additional challenges for data access, interoperability, and cross‐service collaboration. These contextual features may differ in other jurisdictions, affecting transferability of the findings. Further, while the multilevel coding framework offered useful analytic structure, the boundaries between micro‐, meso‐, and macrolevel influences were sometimes difficult for participants to distinguish, and these classifications should be interpreted as heuristic rather than definitive. While patterns in barriers, enablers, and strategies became consistent across participants by the third cohort, suggesting qualitative saturation was approached, the modest absolute sample size (26 entry, 23 exit) means findings should be replicated across other programs and jurisdictions before broader generalizability is claimed. The absence of quantitative outcome data is a limitation; future work combining qualitative implementation data with standardized outcome measures (such as employment trajectories, project outputs, or organizational‐level metrics) would more fully capture the impact of fellowship programs on LHS capability building [20].

Finally, the study relied on self‐reported experiences without observational or documentary data, which may have provided additional insight into organizational processes. Despite these limitations, the study offers valuable insight into how participants navigated multilevel influences during LHS project implementation.

## 5 Conclusion

This study identifies a key misalignment between individual, organizational, and system readiness for LHS project implementation in Australian healthcare. While participants quickly developed confidence and skills, they perceived organizational culture, infrastructure, and policy as changing far more slowly and as constraining project success. Relying on individual champions without corresponding organizational and system investment risks frustration, loss of momentum, and limited progress.

Advancing LHS and broader digital transformation agendas will require deliberate organizational change, including strengthened digital infrastructure, better data interoperability, and supportive governance. Although participatory, locally led projects can generate early enthusiasm, sustained and scalable impact depends on coordinated action across all levels, including committed leadership, adequate infrastructure, workforce capability, and system‐level reform. Alignment across these levels is essential to realize the full potential of LHSs in Australia.

## Funding

This work was supported by the Duncan Leary Bequest.

## Conflicts of Interest

The authors declare no conflicts of interest.

## Supporting information


**Supporting Information 1:** Entry and exit interview guides.

**Supporting Information 2:** The learning health systems academy.

## Data Availability

The data that support the findings of this study are available from the corresponding author upon reasonable request.

## References

[1] C. G. Bocean and A. A. Vărzaru , “Health Status in the Era of Digital Transformation and Sustainable Economic Development,” BMC Health Services Research 25, no. 1 (2025): 343. 40045359 10.1186/s12913-025-12498-y PMC11883919

[2] D. Fleming and S. Chamberlin , Surviving the Future: Culture, Carnival and Capital in the Aftermath of the Market Economy (Chelsea Green Publishing, 2016).

[3] C. P. Friedman , S. M. Greene , and J. C. Rubin , “Ten Reasons Why Learning Health Systems Will Have a Transformational Effect on Health and Health Care,” Learning Health Systems 9, no. 4 (2025): e70044. 41169648 10.1002/lrh2.70044 PMC12569434

[4] J. Braithwaite , K. Churruca , J. C. Long , L. A. Ellis , and J. Herkes , “When Complexity Science Meets Implementation Science: A Theoretical and Empirical Analysis of Systems Change,” BMC Medicine 16, no. 1 (2018): 63. 29706132 10.1186/s12916-018-1057-z PMC5925847

[5] L. A. Ellis , G. Fisher , M. Saba , et al., “Charting Progress in Learning Health Systems: A Systematic Review of 5 Years of Definitions, Models, and Frameworks,” Learning Health Systems 9, no. 3 (2025): e70006. 40677610 10.1002/lrh2.70006 PMC12264394

[6] H. C. Lim , J. A. Austin , A. H. van der Vegt , et al., “Toward a Learning Health Care System: A Systematic Review and Evidence‐Based Conceptual Framework for Implementation of Clinical Analytics in a Digital Hospital,” Applied Clinical Informatics 13, no. 2 (2022): 339–354. 35388447 10.1055/s-0042-1743243 PMC8986462

[7] M. Somerville , C. Cassidy , J. A. Curran , C. Johnson , D. Sinclair , and A. Elliott Rose , “Implementation Strategies and Outcome Measures for Advancing Learning Health Systems: A Mixed Methods Systematic Review,” Health Research Policy and Systems 21, no. 1 (2023): 120. 38012681 10.1186/s12961-023-01071-w PMC10680228

[8] L. A. Ellis , M. Sarkies , K. Churruca , et al., “The Science of Learning Health Systems: Scoping Review of Empirical Research,” JMIR Medical Informatics 10, no. 2 (2022): e34907. 35195529 10.2196/34907 PMC8908194

[9] O. Golburean , E. S. Nordheim , A. Faxvaag , R. Pedersen , O. Lintvedt , and L. Marco‐Ruiz , “A Systematic Review and Proposed Framework for Sustainable Learning Healthcare Systems,” International Journal of Medical Informatics 192 (2024): 105652. 39423652 10.1016/j.ijmedinf.2024.105652

[10] J. Enticott , A. Johnson , and H. Teede , “Learning Health Systems Using Data to Drive Healthcare Improvement and Impact: A Systematic Review,” BMC Health Services Research 21 (2021): 1–16. 33663508 10.1186/s12913-021-06215-8 PMC7932903

[11] P. L. McDonald , T. J. Foley , R. Verheij , et al., “Data to Knowledge to Improvement: Creating the Learning Health System,” BMJ 384 (2024): e076175. 38272498 10.1136/bmj-2023-076175 PMC10809034

[12] C. P. Friedman and S. M. Greene , “Four Distinct Models of Learning Health Systems: Strength Through Diversity,” Learning Health Systems 9, no. 2 (2025): e70009. 40247907 10.1002/lrh2.70009 PMC12000755

[13] M. Menear , M.‐A. Blanchette , O. Demers‐Payette , and D. Roy , “A Framework for Value‐Creating Learning Health Systems,” Health Research Policy and Systems 17, no. 1 (2019): 1–13. 31399114 10.1186/s12961-019-0477-3 PMC6688264

[14] R. J. Reid , W. P. Wodchis , K. Kuluski , et al., “Actioning the Learning Health System: An Applied Framework for Integrating Research Into Health Systems,” SSM—Health Systems 2 (2024): 100010.

[15] G. Fisher , M. Saba , G. Dammery , et al., “Barriers and Facilitators to Learning Health Systems in Primary Care: A Framework Analysis,” BMJ Health & Care Informatics 31, no. 1 (2024): e100946. 10.1136/bmjhci-2023-100946 PMC11328652 38909995

[16] J. Enticott , S. Braaf , A. Johnson , A. Jones , and H. J. Teede , “Leaders' Perspectives on Learning Health Systems: A Qualitative Study,” BMC Health Services Research 20, no. 1 (2020): 1087. 33243214 10.1186/s12913-020-05924-w PMC7689994

[17] Y. Zurynski , C. L. Smith , A. Vedovi , et al., Mapping the Learning Health System: A Scoping Review of Current Evidence—A White Paper (Australian Institute for Health Innovation and the NHMRC Partnership Centre for Health System Sustainability, 2020).

[18] T. Foley and L. Vale , “A Framework for Understanding, Designing, Developing and Evaluating Learning Health Systems,” Learning Health Systems 7, no. 1 (2022): e10315. 36654802 10.1002/lrh2.10315 PMC9835047

[19] S. McLachlan , K. Dube , O. Johnson , et al., “A Framework for Analysing Learning Health Systems: Are We Removing the Most Impactful Barriers?,” Learning Health Systems 3, no. 4 (2019): e10189. 31641685 10.1002/lrh2.10189 PMC6802533

[20] S. Dushyanthen , M. Perrier , W. Chapman , M. Layton , and K. Lyons , “Fostering the Use of Learning Health Systems Through a Fellowship Program for Interprofessional Clinicians,” Learning Health Systems 6, no. 4 (2022): e10340. 36263261 10.1002/lrh2.10340 PMC9576228

[21] L. Shaw , M. Perrier , K. Tahmasebian , et al., “Developing a Codebook to Characterize Barriers, Enablers, and Strategies for Implementing Learning Health Systems From a Multilevel Perspective,” Learning Health Systems 9, no. 2 (2025): e10452. 40247906 10.1002/lrh2.10452 PMC12000758

[22] T. Groenewald , “A Phenomenological Research Design Illustrated,” International Journal of Qualitative Methods 3, no. 1 (2004): 42–55.

[23] J. S. Ancker , N. C. Benda , M. Reddy , K. M. Unertl , and T. Veinot , “Guidance for Publishing Qualitative Research in Informatics,” Journal of the American Medical Informatics Association 28, no. 12 (2021): 2743–2748. 34537840 10.1093/jamia/ocab195 PMC8633663

[24] U. Bronfenbrenner , “Toward an Experimental Ecology of Human Development,” American Psychologist 32, no. 7 (1977): 513–531.

[25] L. J. Damschroder , D. C. Aron , R. E. Keith , S. R. Kirsh , J. A. Alexander , and J. C. Lowery , “Fostering Implementation of Health Services Research Findings Into Practice: A Consolidated Framework for Advancing Implementation Science,” Implementation Science 4 (2009): 1–15. 19664226 10.1186/1748-5908-4-50 PMC2736161

[26] C. B. Forrest , F. D. Chesley, Jr. , M. L. Tregear , and K. B. Mistry , “Development of the Learning Health System Researcher Core Competencies,” Health Services Research 53, no. 4 (2018): 2615–2632. 28777456 10.1111/1475-6773.12751 PMC6051975

[27] A. Assarroudi , F. Heshmati Nabavi , M. R. Armat , A. Ebadi , and M. Vaismoradi , “Directed Qualitative Content Analysis: The Description and Elaboration of Its Underpinning Methods and Data Analysis Process,” Journal of Research in Nursing 23, no. 1 (2018): 42–55. 34394406 10.1177/1744987117741667 PMC7932246

[28] M. D. PL , J. Phillips , K. Harwood , J. Maring , and P. J. van der Wees , “Identifying Requisite Learning Health System Competencies: A Scoping Review,” BMJ Open 12, no. 8 (2022): e061124. 10.1136/bmjopen-2022-061124 PMC9403130 35998963

[29] C. Haraldseid‐Driftland , S. Billett , V. Guise , et al., “The Role of Collaborative Learning in Resilience in Healthcare—A Thematic Qualitative Meta‐Synthesis of Resilience Narratives,” BMC Health Services Research 22, no. 1 (2022): 1091. 36028835 10.1186/s12913-022-08451-y PMC9412809

[30] D. Easterling , A. C. Perry , R. Woodside , T. Patel , and S. B. Gesell , “Clarifying the Concept of a Learning Health System for Healthcare Delivery Organizations: Implications From a Qualitative Analysis of the Scientific Literature,” Learning Health Systems 6, no. 2 (2022): e10287. 35434353 10.1002/lrh2.10287 PMC9006535

[31] D. M. Nash , Z. Bhimani , J. Rayner , and M. Zwarenstein , “Learning Health Systems in Primary Care: A Systematic Scoping Review,” BMC Family Practice 22, no. 1 (2021): 126. 34162336 10.1186/s12875-021-01483-z PMC8223335

[32] Australian Commission on Safety and Quality in Health Care , Improving Clinical Communication, Collaboration and tEAMWORK in Australian Health Services (ACSQHC, 2020).

[33] D. Peiris , A.‐M. Feyer , J. Barnard , et al., “Overcoming Silos in Health Care Systems Through Meso‐Level Organisations–a Case Study of Health Reforms in New South Wales, Australia,” Lancet Regional Health—Western Pacific 44 (2024): 101013. 38384947 10.1016/j.lanwpc.2024.101013 PMC10879775

[34] B. Page , D. Irving , R. Amalberti , and C. Vincent , “Health Services Under Pressure: A Scoping Review and Development of a Taxonomy of Adaptive Strategies,” BMJ Quality and Safety 33, no. 11 (2024): 738–747. 10.1136/bmjqs-2023-016686 PMC11503202 38050158

[35] T. Blake , D. Passey , J. Lee , and F. Rizvi , “Assessing the Digital Health Maturity of General Practice in Australia: Results From a Cross‐Sectional National Survey,” Australian Journal of Primary Health 31, no. 5 (2025): PY25107. 40977214 10.1071/PY25107

[36] K. Cresswell , F. Jahn , L. Silsand , et al., “Assessing Digital Maturity of Hospitals: Viewpoint Comparing National Approaches in Five Countries,” Journal of Medical Internet Research 27 (2025): e57858. 40053724 10.2196/57858 PMC11926443

[37] Victorian Department of HaHS , Victorian Public Health Services Digital Health Maturity Assessment: Summary Report 2019 (Victorian Government, 2019).

[38] C. P. Friedman , E. A. Lomotan , J. E. Richardson , and J. L. Ridgeway , “Socio‐Technical Infrastructure for a Learning Health System,” Learning Health Systems 8, no. 1 (2024): e10405. 38249851 10.1002/lrh2.10405 PMC10797563

[39] S. Witter , K. Sheikh , and M. Schleiff , “Learning Health Systems in Low‐Income and Middle‐Income Countries: Exploring Evidence and Expert Insights,” BMJ Global Health 7, no. Suppl 7 (2022): e008115. 10.1136/bmjgh-2021-008115 PMC9490579 36130793

[40] D. Rajit , A. Johnson , E. Callander , H. Teede , and J. Enticott , “Learning Health Systems and Evidence Ecosystems: A Perspective on the Future of Evidence‐Based Medicine and Evidence‐Based Guideline Development,” Health Research Policy and Systems 22, no. 1 (2024): 4. 38178086 10.1186/s12961-023-01095-2 PMC10768258

[41] S. PAD , G. Wardi , R. A. Harrington , and C. A. Longhurst , “Learning Health System Strategies in the AI Era,” npj Health Systems 2, no. 1 (2025): 21. 10.1038/s44401-025-00029-0 PMC13354208 42527455

