# Supplementary material for: Exploring Barriers, Enablers, and Strategies for Implementing Learning Health System Projects in Healthcare Organizations: A Multilevel Analysis in an Australian Setting
Source: Learn Health Syst. 2026 Jul 13;10(3):e70103. doi: 10.1002/lrh2.70103 (PMC13364508; doi:10.1002/lrh2.70103)
Supplement: Supplementary file 1 — Supporting Information 1: Entry and exit interview guides. [file LRH2-10-e70103-s001.docx]

**Supplemental material 1: Entry and exit interview guides**

**Entry interview guide**

**1a. Let’s start with the individual level, what barriers have you experienced within yourself that has affected your LHS project?**

[if prompts required]

- Leadership culture of learning
- Time constraints
- Supportive system
- Program design
- Level in organisation
- Working environment
- Working styles
- Personality traits

**1b. What outcomes have these had on your project to date (if any)?**

**1c. What strategies have you used to overcome these barriers?**

[if prompts required – to consider]

- Utilisation of HR
- Networking
- Seeking support
- Improved time management
- Encouraging participant engagement

**2. On an individual level, what strengths (or enablers) in yourself have you noticed that have positively affected your project?**

**2a. Have you surprised yourself in any way? Anything unexpected?**

[If prompts required]

- HR
- Participants’ engagement
- Ability to work flexibly
- Interpersonal relationships
- Personality traits

**3. At a departmental or organisational level, what barriers have you experienced that have affected your project?**

[if prompts required, consider]

- Data quality
- Gaps in digital maturity
- Interoperability of systems
- Incentives for value
- Leadership instilled culture
- Time constraints
- Complexity of illnesses
- Internal communication

**4. At a departmental or organisational level, how has your dept or org positively affected the project?**

[if prompt required, consider] E.g.

- Digital capture of care experience
- Engaged colleagues
- Engaged patients
- Leadership culture
- Data -acquisition, aggregation

**ONLY If time permits:**

How has your organisations’ digital maturity affected the ability for your project to be adopted?

Prompts:

Has a digital maturity assessment been conducted on your organisation that you are aware of?

Is there a need for a digital maturity assessment?

How ready is your organisation to adopt a digitally enabled LHS model?

Prompts:

How does your organisation approach change management?

How does your organisation approach digital adoption?

How does your organisation integrate learnings into improved actions?

##

**Exit interview guide**

**1a. Reflecting back on your project what were the main barriers you experienced within yourself that affected your LHS project?**

[if prompts required]

- Leadership culture of learning
- Time constraints
- Supportive system
- Program design
- Level in organisation
- Working environment
- Working styles
- Personality traits

Anything from LADs OR Entry Interview (bullet point) – do you wish to expand or add onto any of these?

**1b. What outcomes did these have on your project?**

**1c. What strategies did you used to overcome these barriers?**

[if prompts required – to consider]

- Utilisation of HR
- Networking
- Seeking support
- Improved time management
- Encouraging participant engagement

**2a. On an individual level, what strengths in yourself have you noticed develop over the year that have positively affected your project?**

**2b. Have you surprised yourself in any way? Anything unexpected?**

[If prompts required]

- HR
- Participants’ engagement
- Ability to work flexibly
- Interpersonal relationships
- Personality traits

**3. What do you see as your role in your health organisation now? How has this changed through the year?**

[if prompts required]

- through the project
- through the program

**4a. At a departmental or organisational level, what barriers throughout the year did you experience that affected your project?**

**4b. What missed opportunities were there? Were there negative results?**

[if prompts required, consider]

- Data quality
- Gaps in digital maturity
- Interoperability of systems
- Incentives for value
- Leadership instilled culture
- Time constraints
- Complexity of illnesses
- Internal communication

**5. At a departmental or organisational level, how has your dept or org positively affected the project? Were there any specific enablers?**

[if prompt required, consider] E.g.

- Consider digital capacity and maturity
- Consider LHS awareness, appetite to adopt as a potential model
- Digital capture of care experience
- Engaged colleagues
- Engaged patients
- Leadership culture
- Data -acquisition, aggregation

**ONLY If time permits:**

How has your organisations’ digital maturity affected the ability for your project to be adopted?

Prompts:

Has a digital maturity assessment been conducted on your organisation that you are aware of?

Is there a need for a digital maturity assessment?

How ready is your organisation to adopt a digitally enabled LHS model?

Prompts:

How does your organisation approach change management?

How does your organisation approach digital adoption?

How does your organisation integrate learnings into improved actions?
