# Supplementary material for: Exploring Barriers, Enablers, and Strategies for Implementing Learning Health System Projects in Healthcare Organizations: A Multilevel Analysis in an Australian Setting
Source: Learn Health Syst. 2026 Jul 13;10(3):e70103. doi: 10.1002/lrh2.70103 (PMC13364508; doi:10.1002/lrh2.70103)
Supplement: Supplementary file 2 — Supporting Information 2: The learning health systems academy. [file LRH2-10-e70103-s002.pptx]

## Slide 1
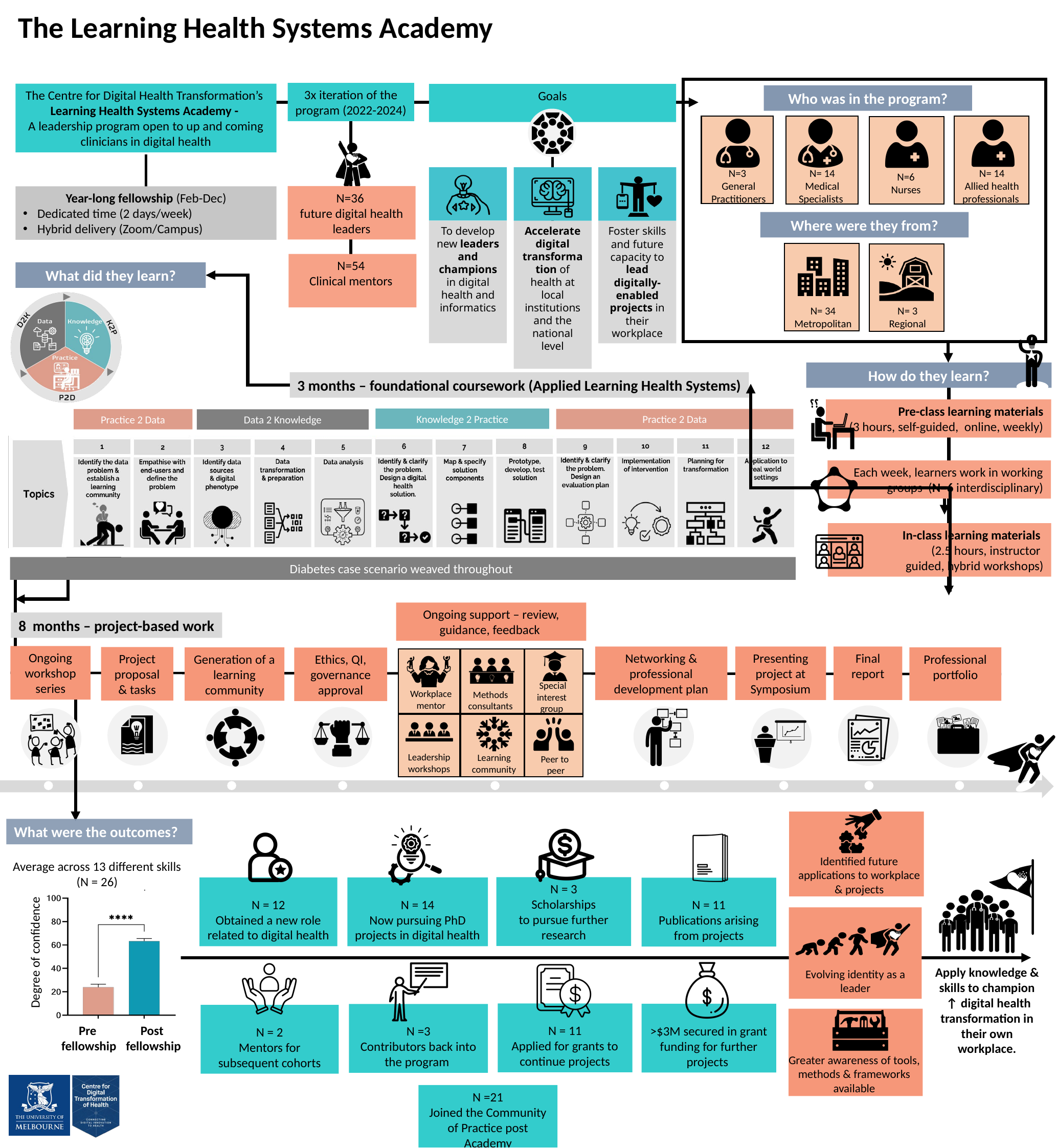

The Learning Health Systems Academy
Who was in the program?
3x iteration of the program (2022-2024)
The Centre for Digital Health Transformation’s
Learning Health Systems Academy -
A leadership program open to up and coming clinicians in digital health
Goals
N= 14
Allied health professionals
N= 14
Medical Specialists
N=3
General Practitioners
N=6
Nurses
To develop new leaders and champions in digital health and informatics
Accelerate digital transformation of health at local institutions and the national level
Foster skills and future capacity to lead digitally-enabled projects in their workplace
N=36
future digital health leaders
Year-long fellowship (Feb-Dec)
Dedicated time (2 days/week)
Hybrid delivery (Zoom/Campus)
Where were they from?
N=54
Clinical mentors
What did they learn?
N= 3
Regional
N= 34
Metropolitan
How do they learn?
3 months – foundational coursework (Applied Learning Health Systems)
Pre-class learning materials
(3 hours, self-guided, online, weekly)
Knowledge 2 Practice
Practice 2 Data
Practice 2 Data
Data 2 Knowledge
Diabetes case scenario weaved throughout
Each week, learners work in working groups (N=6 interdisciplinary)
In-class learning materials
(2.5 hours, instructor
guided, hybrid workshops)
Ongoing support – review, guidance, feedback
8 months – project-based work
Ongoing workshop series
Presenting project at Symposium
Final report
Networking & professional development plan
Project proposal
& tasks
Generation of a learning
 community
Professional portfolio
Ethics, QI, governance
 approval
Methods consultants
Special interest
group
Workplace mentor
Peer to
 peer
Leadership workshops
Learning community
Identified future applications to workplace & projects
What were the outcomes?
Average across 13 different skills (N = 26)
Degree of confidence
Post
fellowship
N = 3
Scholarships
to pursue further research
N = 12
Obtained a new role related to digital health
N = 14
Now pursuing PhD projects in digital health
N = 11
Publications arising from projects
Evolving identity as a leader
Apply knowledge & skills to champion
 ↑ digital health transformation in their own workplace.
N = 11
Applied for grants to continue projects
>$3M secured in grant funding for further projects
N =3
Contributors back into the program
N = 2
Mentors for subsequent cohorts
Greater awareness of tools, methods & frameworks available
Pre
fellowship
N =21
Joined the Community of Practice post Academy
